# Comparison of patient outcomes in periarticular and intraarticular local anaesthetic infiltration techniques in total knee arthroplasty

**DOI:** 10.1186/s13018-015-0249-x

**Published:** 2015-07-31

**Authors:** Michael Perret, Philip Fletcher, Laura Firth, Piers Yates

**Affiliations:** Department of Orthopaedics, Fremantle Hospital, Alma St, Fremantle, WA 6160 Australia; Sir Charles Gairdner Hospital, Hospital Ave, Nedlands, WA 6009 Australia; Centre for Applied Statistics, University of Western Australia, 35 Stirling Hwy, Crawley, WA 6009 Australia; University of Western Australia, Fremantle, WA 6160 Australia; Fiona Stanley Hospital, Fremantle, WA 6160 Australia; Fremantle Hospital, Fremantle, WA 6160 Australia

**Keywords:** Local infiltration analgesia, Local anaesthetic, Total knee arthroplasty, Total knee replacement

## Abstract

**Background:**

The use of local infiltration analgesia in the setting of knee arthroplasty is well established. There are no studies to date which have directly compared differences in infiltration techniques. The purpose of this study is to establish if a difference in patient outcomes exists when the infiltrate is injected into the periarticular tissues or directly into the joint.

**Methods:**

One hundred and forty-two consecutive patients waitlisted for primary total knee arthroplasty were enrolled after primary exclusion criteria were applied. These included the following: allergy to study drugs, inability to receive spinal anaesthesia, and planned bilateral surgery. Patients were divided into two groups, a periarticular infiltration group (group A) and an intraarticular infiltration group (group B). Secondary exclusion criteria of regular opioid use, psychiatric illness, and serious medical comorbidity left a total of 47 patients in group A and 54 patients in group B. Both groups received a combination of 30 mg ketorolac, 500 μg of adrenaline, and 300 mg of ropivacaine, and normal saline. This was either injected into the periarticular tissues during surgery (group A) or intraarticularly after closure of the wound (group B).

Primary outcome measures included opioid consumption during the first 24 h postoperatively and over the total admission, and visual analogue scales (VAS) on postoperative day 1 and at discharge. Secondary measures included Oxford Knee Score, knee flexion, length of stay, haemoglobin drop, and transfusion requirement.

Ethics approval was granted by the hospital review board. The trial is registered in the Australian New Zealand Clinical Trials Registry, registration ACTRN12615000488505.

**Results:**

No statistically significant differences in postoperative analgesic use were observed between the two groups. However, there was a trend toward decreased postoperative patient-controlled analgesia use in the periarticular group (mean 53.1 vs 68.3 mg morphine equivalents; *p* = 0.093), as well as a statistically significant reduction in postoperative visual analogue pain scores. No statistically significant differences were observed for haemoglobin drop, range of motion, or pre- to 6-week postoperative Oxford Score difference.

**Conclusions:**

Our study is the first we are aware of to directly compare a periarticular to intraarticular injection technique when using local infiltration analgesia for total knee arthroplasty. Our results show no clear statistically significant benefit with either technique. The periarticular group showed a statistically significant reduction in postoperative VAS pain scores alongside a trend in that group toward reduced overall opioid use.

## Background

Total knee arthroplasty for osteoarthritis is a common procedure, with over 50,000 procedures performed each year in Australia [1]. Demand for the operation is increasing as the population continues to age and expand. The procedure is associated with significant postoperative pain, and interest has grown in local infiltration analgesia (LIA) which has gained popularity in part due to the side effects of traditional systemic opioid-based regimens.

Kerr and Kohan [2] developed a LIA technique in which ropivacaine, ketorolac, and adrenaline are infiltrated into the periarticular tissues at the time of surgery, and also postoperatively via an intraarticular catheter. The efficacy of LIA techniques in the setting of total knee arthroplasty has been supported by multiple studies in the literature [3–7].

Several modifications to the technique described by Kerr and Kohan have been published, including alterations in the composition of infiltrated agents, their concentrations and volumes, the timing and location of infiltration, and the use of intraarticular catheters for postoperative infusion. In the interests of refining our LIA technique, we undertook a literature review and found that there were no studies which directly compared a periarticular to an intraarticular injection technique.

The purpose of our study was to investigate the difference in outcomes of two separate methods of administering LIA, namely intraarticular and periarticular tissue infiltration. Primary measures investigated were related to analgesic efficacy, measured by postoperative opioid consumption and visual analogue pain scores. Secondary endpoints included an assessment of functional outcome in terms of the Oxford Knee Score, length of stay (LOS), range of motion (ROM), and postoperative haemoglobin (Hb) drop as an indirect measure of blood loss.

## Methods

Ethics approval was granted by the hospital research and ethics committee.

We identified patients scheduled for primary total knee arthroplasty for osteoarthritis. Patients were enrolled from a public hospital orthopaedic outpatient clinic between April 2008 and January 2012, and were allocated to either the periarticular infiltration group (group A) or the intraarticular group (group B) depending on the date of their surgery. All patients gave their informed consent to participate. Seventy-one patients were identified in the first 18 months of the study period and were assigned to group A. The following 71 patients were allocated to group B, giving a total of 142 consecutive patients. Trial exclusion criteria were allergy or intolerance to study drugs, known inability to receive spinal anaesthesia, and planned bilateral knee surgery.

A retrospective review of medical records identified patients with a history of at least once-daily opioid use, concurrent active psychiatric illness (not including controlled depression/anxiety), serious medical comorbidities that might prolong hospital admission, and coagulation disorders, and these patients were also excluded from our analysis.

All surgery was performed under spinal anaesthesia by a consultant surgeon and/or his fellow using the same technique as described below:

A standard medial parapatellar approach was used. A tourniquet was inflated at the start of the procedure and deflated after skin closure. One gram of tranexamic acid was given orally 1 h before surgery and 6 h after. Two grams of intravenous cephazolin was given at induction.

Patients all received a PFC Sigma RP posterior stabilised (Depuy, Warsaw, IN) prosthesis, which was cemented with Palacos with gentamycin cement (Heraeus Kulzer, Hanau, Germany). The patella was resurfaced on all patients.

A combination of 30 mg of ketorolac (1 mL), 500 μg of adrenaline (5 mL of 1:10,000), and 300 mg of ropivacaine (40 mL of 0.75 %) was added to normal saline to create a total volume of 150 mL. This was based on the regime described by Kerr and Kohan [2].

In group A patients, the 150 mL was infiltrated after implantation of the prosthesis, prior to insertion of the polyethylene liner. Of this, 50 mL was infiltrated into the posterior capsule and intercondylar area; 50 mL was infiltrated into the anterior capsule, the collateral ligaments, and along the femur and tibia; and the remaining 50 mL was infiltrated into subcutaneous tissue following closure of the capsule. Group B patients had all of the 150 mL injected intraarticularly after closure of the wound. No drains were inserted.

Postoperatively, all patients received paracetamol 1 g 6-hourly and the use of a patient-controlled analgesia (PCA) device with fentanyl (10 μg bolus, 6 min lockout) or morphine (1 mg bolus, 6 min lockout). The PCA was discontinued after clinical review by the anaesthetist, usually 24 h postoperatively. Rescue oral analgesia in the form of oxycodone IR (immediate release) or tramadol was administered as required throughout the course of the admission. All patients received 40 mg enoxaparin daily for deep vein thrombosis prophylaxis commencing the following morning.

Mobilisation was attempted on the first postoperative day under the supervision of a physiotherapist, and a standardised rehabilitation programme was delivered to the patients. This involved twice-daily sessions with the physiotherapist until the patients were able to independently mobilise with a gait aid, perform independent transfers, and undertake basic activities of daily living. Patients were discharged home once these criteria were fulfilled, their pain was adequately controlled with oral analgesia, and there was no evidence of surgical complication.

### Primary outcomes

The primary outcome measures were opioid consumption during the first 24 h, total opioid consumption over admission, and visual analogue scales (VAS) on postoperative day 1 and at discharge. Opioid doses were converted into morphine equivalents [8].

### Secondary outcomes

Oxford Knee Scores were calculated preoperatively and 6 weeks postoperatively. Knee flexion was measured preoperatively, at discharge, and 6 weeks postoperatively. Length of stay was noted. Preoperative and postoperative haemoglobin measurements on postoperative day 1 were recorded as a marker for operative blood loss, as was any requirement for blood transfusion. Requirement for transfusion was determined by our local hospital guidelines of symptomatic anaemia (associated with increased oxygen requirement or cardiorespiratory decompensation) or active bleeding.

### Statistics

Differences between groups A and B for PCA usage, total opioid analgesia, Oxford Score, range of motion, haemoglobin drop, VAS at day 1 and discharge, and LOS were analysed using a *t* test assuming non-equal variance. Boxplots of each variable were also created to detect possible outliers.

PCA usage, total opioid analgesia (24 h), and total opioid analgesia (over admission) were also assessed in a multiple regression model to see if there was a difference between groups A and B after adjusting for age, Oxford Score (pre- and postoperatively), and ROM (pre- and postoperatively). Square root transformations were used for PCA usage and both measures of total opioid analgesia to correct for violations of the constant variance assumption for regression.

## Results

A total of 142 patients were enrolled into our study. Of these, 41 were secondarily excluded, most commonly for history of regular opioid use. Where incomplete data were identified, the number of patients excluded is shown in Table 1.

**Table 1:** Data comparison between group A and group B

Variable	Group A		Group B		*p* value
	Mean (sd)	*n*	Mean (sd)	*n*	
Age	68.8 (9.8)	47	66.2 (9.4)	54	0.1717
PCA	53.1 (41.0)	47	68.3 (49.3)	54	0.0934
Total opioid analgesia (24 h)	58.7 (45.8)	47	72.4 (50.7)	54	0.1667
Total opioid analgesia (admission)	118.5 (109.5)	47	125.7 (74.9)	54	0.7060
Oxford (pre)	41.1 (5.5)	47	40.8 (5.9)	51	0.8053
Oxford (6 weeks post)	20.9 (6.6)	42	24.2 (5.4)	41	0.0143*
Oxford (difference)	−19.9 (7.9)	42	−16.8 (8.0)	41	0.0869
ROM (pre)	111.1 (17.1)	47	112.1 (14.6)	54	0.7398
ROM (6 weeks post)	110.8 (12.8)	47	109.7 (10.2)	54	0.6309
ROM (difference)	−0.30 (15.9)	47	−2.48 (15.5)	54	0.4885
Haemoglobin (difference)	26.4 (8.6)	45	24.2 (8.4)	51	0.2130
VAS day 1	3.83 (2.0)	47	4.63 (1.8)	54	0.0391*
VAS discharge	2.47 (1.6)	47	2.87 (1.5)	54	0.2014
VAS discharge without outliers	2.24 (1.2)	47	2.87 (1.5)	54	0.0253*
LOS	4.30 (2.1)	47	3.44 (0.7)	54	0.0104*
LOS without outliers	3.86 (1.2)	47	3.44 (0.7)	54	0.0386*

*p value of <0.05

The number of patients in group A was 47 and group B comprised 54 patients. Patient ages were similarly matched.

### Primary outcome measures

#### Opioid consumption

**Fig. 1:**
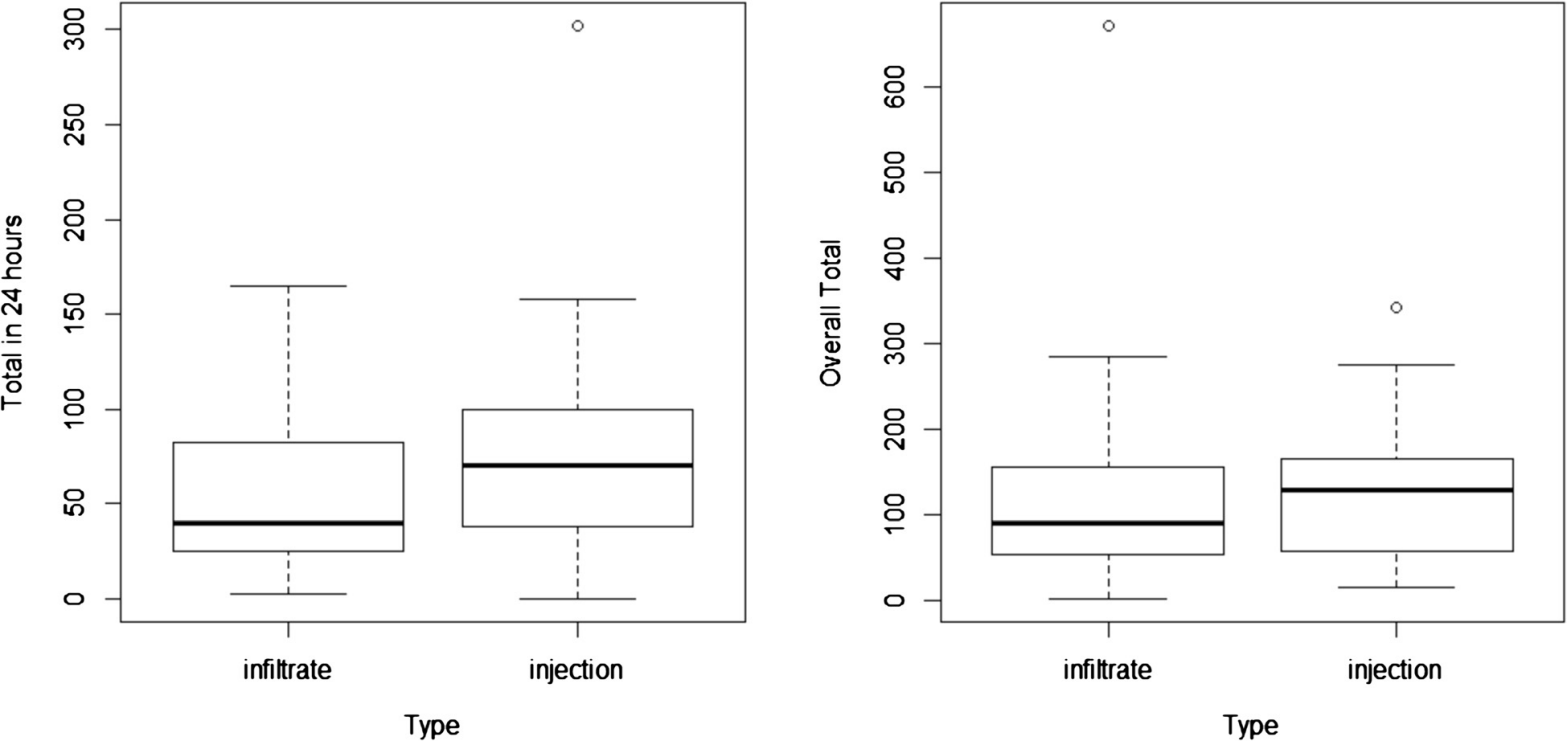
Opioid usage in the first 24 h postoperatively and over total admission, comparing infiltrate (group A) vs injection (group B)

Mean values for the primary endpoints of PCA use, total opioid analgesia (24 h), and total opioid analgesia (admission) were lower in group A than in group B, though these differences were not statistically significant (Fig. 1).

Plots of PCA use over 24 h postoperatively, total opioid analgesia use over 24 h postoperatively, and total opioid analgesia over admission indicated possible outliers. When these were removed, the difference between groups A and B was still not significant (*p* values = 0.1739, 0.2950, and 0.1980, respectively).

#### VAS

**Fig. 2:**
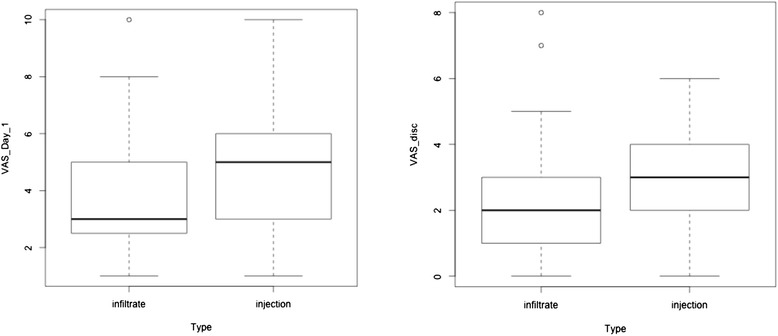
VAS at postoperative day 1 and at discharge, comparing infiltrate (group A) vs injection (group B)

VAS scores in group A were significantly lower than those in group B at postoperative day 1 (3.83 vs 4.63; *p* value = 0.039). VAS scores in group A were also lower than those in group B at discharge, though this difference was not significant (Fig. 2). Boxplots for VAS at discharge indicated possible outliers, and the difference between group A and group B reached statistical significance once these outliers were removed (*p* value = 0.0253).

### Secondary outcome measures

#### Length of stay

The LOS data demonstrated a significantly higher LOS in group A patients (4.30 vs 3.44 days; *p* value = 0.0104). The boxplots indicated three possible outliers: two with LOS of 9 days and one with 14 days. These prolonged admissions were a result of medical complications. When these outliers are removed, the difference in LOS is smaller but still statistically significant (*p* value = 0.0386).

#### Oxford Scores

**Fig. 3:**
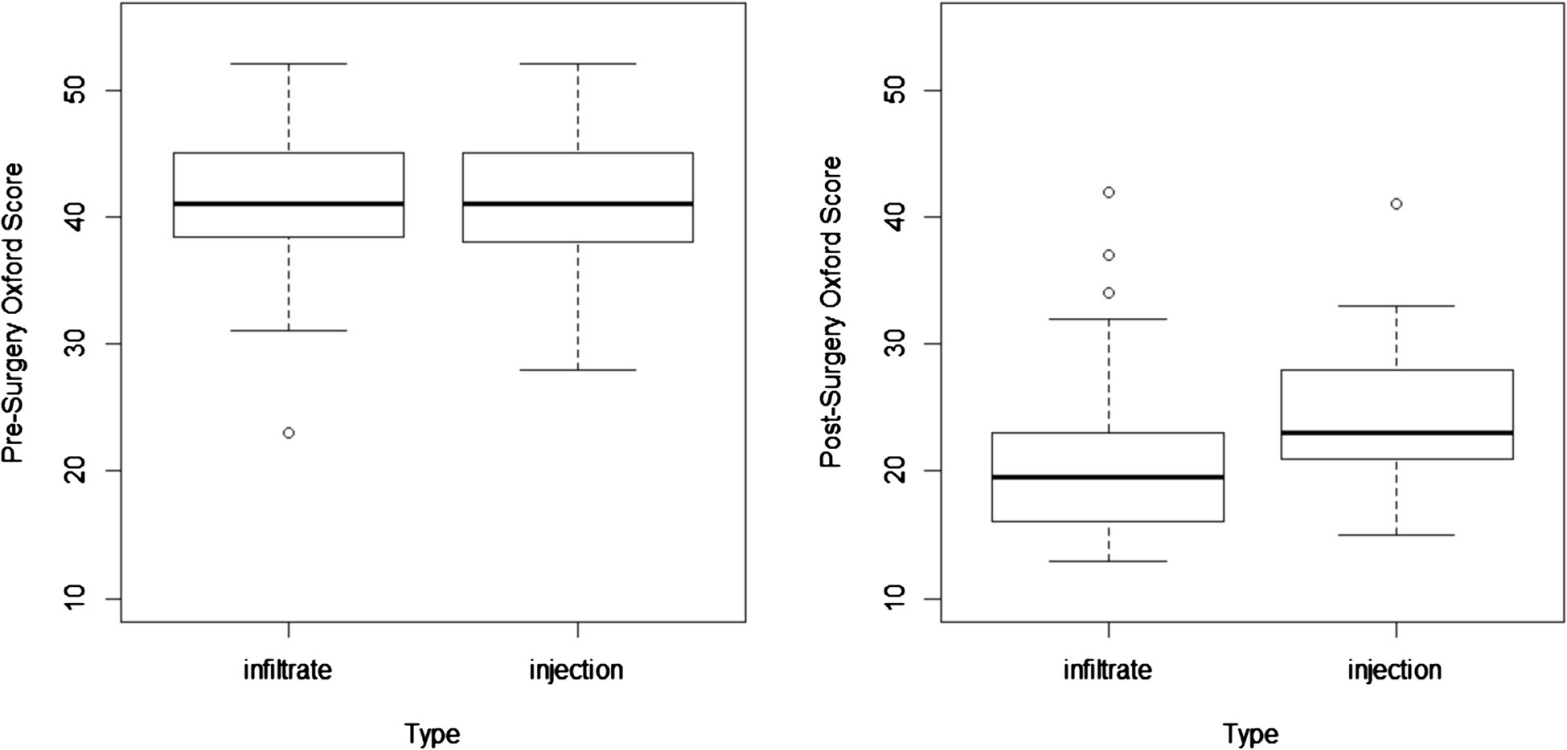
Oxford Knee Scores preoperatively and at 6 weeks postoperatively, comparing infiltrate (group A) vs injection (group B)

The groups were similar in terms of preoperative Oxford Scores; however, the 6-week postoperative Oxford Scores were different, with group B having a significantly better (higher) Oxford Score than group A (24.2 vs 20.9; *p* value = 0.0143). This statistically significant effect disappeared when the scores were compared to preoperative scores (Fig. 3).

#### Knee range of motion

No statistically significant difference was demonstrated between groups.

#### Haemoglobin drop

There was minimal difference between the groups when postoperative haemoglobin drop was examined and there was no statistical significance. There were a total of four transfusions recorded (two in each group).

### Multiple regression analysis

PCA, total opioid analgesia (24 h), and total opioid analgesia (admission) were assessed in a multiple regression model to examine if there was a difference between groups A and B after adjusting for age, Oxford Score (pre- and postoperative), and ROM (pre- and postoperative) (Table 2).

**Table 2:** Multiple regression results—significant variables only

Response variable	Independent variables	Coefficient	*p* value
Sqrt (PCA)	Age	−0.089	0.0104
	Oxford (post)	0.145	0.0057
Sqrt (total opioid analgesia [24 h])	Age	−0.107	0.0038
	Oxford (post)	0.163	0.0080
Sqrt (total opioid analgesia [admission])	Age	−0.165	0.0001
	Oxford (pre)	0.177	0.0092
	Oxford (post)	0.160	0.0096

The initial analysis indicated a problem with the variance of the residuals, so a square root transformation was used for the response variable. The results indicated that after adjusting for these variables, there was still no difference between groups A and B as this variable indicating treatment group was not significant. The models were initially fitted with group, age, preoperative Oxford Score, postoperative Oxford Score, and pre- and postoperative ROM, and subsequently the non-significant variables were removed.

## Discussion

The role of LIA in the setting of total knee arthroplasty has been established in the literature. The goal of our study was to determine any difference in primary and secondary outcome measures when periarticular infiltration was compared to intraarticular injection at the time of surgery. To our knowledge, there have been no published studies examining this.

The techniques for LIA and in particular the target tissues vary in the literature. It is still unclear which tissues are responsible for generating pain in the setting of total knee arthroplasty. Periarticular infiltration techniques target the joint capsule, deep tissues surrounding the collateral ligaments, and the subcutaneous tissues and wound edges [2]. Anderson et al. [3] found that ropivacaine infiltrated into the subcutaneous tissues intraoperatively was a key component in postoperative pain control. The study also concluded that ropivacaine boluses administered via a catheter placed in the subcutaneous tissues 24 h postoperatively were ineffective and of no benefit in terms of patient analgesia.

Several studies have examined the efficacy of intraarticular infiltration, both intraoperatively and postoperatively. Badner et al. [6] found that intraarticular injections of bupivacaine and adrenaline at wound closure reduced the postoperative need for opiates in total knee arthroplasty. These results were not supported by a subsequent larger study by Ritter et al. [7]. A study of 60 patients undergoing total knee arthroplasty published in 2004 [5] found that a bupivacaine bolus injected intraarticularly at capsule closure was associated with reduced opioid consumption, though this result was not statistically significant. None of these studies found a statistically significant reduction in measured pain scores.

The benefit of intraarticular catheters to provide supplementary dosages of local anaesthetic in the postoperative period is unproven. In addition to the lack of evidence surrounding the benefit of ongoing boluses, intraarticular catheter placement is less desirable given the potential for infection. Essving et al. [4] cultured drain tips from 48 patients at 24 h post total knee arthroplasty, with three resultant positive coagulase-negative *Staphylococcus* cultures. No clinically significant infections developed in these patients.

Our results did not show any statistically significant difference between groups in postoperative opioid requirements. The periarticular group showed trends toward reduced PCA usage (53.1 vs 68.3 mg morphine equivalent; *p* = 0.093) and reduced total opioid analgesia requirement within 24 h postoperatively (58.7 vs 72.4 mg; *p* = 0.167). The total opioid analgesic requirement over admission was similar between groups (118.5 mg in group A vs 125.7 mg in group B; *p* = 0.706), though when considered alongside LOS data, this analgesia was spread over a longer length of stay in the periarticular group.

It has been observed in the literature [3–7] that periarticular infiltration is effective in reducing opioid consumption postoperatively. This would suggest that the tissues responsible for generating pain in the setting of total knee arthroplasty may be better targeted by a periarticular technique. The authors of a systematic review of the literature in 2012 [9] advocated delivery by systematic infiltration of all exposed tissues, including the posterior capsule. In our experience, there is a small amount of crossover in techniques, as during periarticular infiltration, there is inevitably a small amount of solution that remains within the joint, and in intraarticular injection, solution may escape into the periarticular tissues.

Interestingly, multiple regression analysis showed that as age increased, the amount of PCA used, total opioid analgesia during the first 24 h, and total opioid analgesia over the course of the admission tended to decrease. The reason for this age-related reduction in analgesic requirement is unclear; it may be related to reduced metabolism of opioids in the elderly or possibly reduced rates of pain reporting in this population.

There was a statistically significant decrease in the VAS scores of patients in the periarticular group when compared to the intraarticular group 24 h postoperatively. This occurred alongside a trend toward reduced early opioid requirements in this group—these patients experienced less pain whilst possibly requiring less opioids. Although the observed reduction in PCA use (53.1 vs 68.3 mg morphine equivalents; *p* = 0.093) fails to reach statistical significance, in combination with reduced VAS scores at 24 h, it lends support to a possible advantage in the periarticular technique. It is also noted that once outliers from the periarticular group are removed, there is a statistically significant decreased VAS on discharge. This is unexpected as the duration of action of LIA agents is far less than our average length of stay (3.9 days).

Another possible explanation for the higher VAS scores observed in the intraarticular injection group relates to the large volume of fluid and subsequent swelling of the knee joint with intraarticular injection. A proportion of this fluid will inevitably escape into the surrounding tissues or be quickly reabsorbed. A similar volume effect may occur to a lesser extent in the periarticular group as blood collects in the joint postoperatively, though the overall volumes are likely to be larger in the periarticular group.

Our measured length of stay data is contradictory to the reduced VAS scores at 24 h and at discharge observed in the periarticular infiltration group, in that the intraarticular group showed a statistically significant shorter length of stay (3.86 vs 3.44 days; *p* = 0.0386, with outliers removed). The reduction in VAS scores at time of discharge in the periarticular group may be partly explained by the fact that these patients had slightly longer to recover from their surgery.

Oxford Knee Scores at 6 weeks postoperatively were observed to be higher in the intraarticular group (24.2 vs 20.9; *p* = 0.0143), though this benefit disappeared once preoperative scores were considered and a difference between pre- and postoperative scores was calculated.

There was no significant difference in the mean haemoglobin drop between the groups. It has been postulated that adrenaline may reduce postoperative bleeding by a vasoconstrictive and tamponade effect. We recognise the shortfalls in measuring haemoglobin as a surrogate marker of local bleeding, and could not account for confounding factors arising from patient comorbidities and surgical technique. Our result is however consistent with the literature, as documented in a systematic review by Gibbs et al. [9].

Other limitations of our study included a lack of randomisation, potential inaccuracy in the standardisation of morphine equivalents, and use of differing analgesic regimes between patients. It would have been beneficial to increase the frequency of VAS measurements and opioid usage especially early in the postoperative period to further stratify any differences.

## Conclusion

We are not aware of any other study that compares targeting different tissues using local infiltration analgesia during total knee arthroplasty. Our results demonstrated no statistically significant difference in our primary endpoint of opioid usage between periarticular and intraarticular groups. Periarticular infiltration was associated with reduced VAS scores at 24 h postoperatively and at discharge (with outliers removed), and a slightly longer length of stay.
